# Protocol: Remote care as the ‘new normal’?  Multi-site case study in UK general practice

**DOI:** 10.3310/nihropenres.13289.1

**Published:** 2022-08-08

**Authors:** Trisha Greenhalgh, Sara E Shaw, Anica Alvarez Nishio, Amy Booth, Richard Byng, Aileen Clarke, Francesca Dakin, Roz Davies, Stuart Faulkner, Nina Hemmings, Laiba Husain, Asli Kalin, Emma Ladds, Lucy Moore, Rebecca Rosen, Sarah Rybczynska-Bunt, Joseph Wherton, Sietse Wieringa

**Affiliations:** 1 Nuffield Department of Primary Care Health Sciences, University of Oxford, Oxford, OX2 6GG, UK; 2 Independent Lay Adviser, London, UK; 3 University of Plymouth, Plymouth, UK; 4 Thrive by Design, Leeds, UK; 5 Nuffield Trust, London, UK; 6 Centre for Sustainable Health Education, University of Oslo, Oslo, Norway

**Keywords:** Remote consultations, general practice, digital inclusion, triage, access, video consultations, telephone consultations, e-consultations

## Abstract

**Background:**

Following a pandemic-driven shift to remote service provision, UK general practices offer telephone, video or online consultation options alongside face-to-face. This study explores practices’ varied experiences over time as they seek to establish remote forms of accessing and delivering care.

**Methods:**

This protocol is for a mixed-methods multi-site case study with co-design and national stakeholder engagement. 11 general practices were selected for diversity in geographical location, size, demographics, ethos, and digital maturity. Each practice has a researcher-in-residence whose role is to become familiar with its context and activity, follow it longitudinally for two years using interviews, public-domain documents and ethnography, and support improvement efforts. Research team members meet regularly to compare and contrast across cases. Practice staff are invited to join online learning events. Patient representatives work locally within their practice patient involvement groups as well as joining an online patient learning set or linking via a non-digital buddy system. NHS Research Ethics Approval has been granted. Governance includes a diverse independent advisory group with lay chair. We also have policy in-reach (national stakeholders sit on our advisory group) and outreach (research team members sit on national policy working groups).

**Results (anticipated):**

We expect to produce rich narratives of contingent change over time, addressing cross-cutting themes including access, triage and capacity; digital and wider inequities; quality and safety of care (e.g. continuity, long-term condition management, timely diagnosis, complex needs); workforce and staff wellbeing (including non-clinical staff, students and trainees); technologies and digital infrastructure; patient perspectives; and sustainability (e.g. carbon footprint).

**Conclusion:**

By using case study methods focusing on depth and detail, we hope to explain why digital solutions that work well in one practice do not work at all in another. We plan to inform policy and service development through inter-sectoral network-building, stakeholder workshops and topic-focused policy briefings.

## Plain English summary

The pandemic required general practices to introduce remote (phone, video and email) consultations. That policy undoubtedly saved lives at the time but there are also clear benefits of face-to-face consultations in some circumstances, and the exact role of remote care still needs to be worked out. Despite best efforts, remote care tends to worsen health inequities (people who were poor or less well educated are less able to access and navigate the system and secure the type of appointment they need or prefer).

Workstream 1: We will look at 11 GP surgeries across England, Scotland and Wales. We have selected a variety of sites: urban and rural, serving a range of different communities. Each surgery has a different approach to technology. A researcher from our team will work alongside surgery staff to learn what methods and technologies each practice uses to deliver care. They will gather information (mostly qualitative) about how different technological solutions are playing out over time.

Workstream 2: Many people experience barriers to accessing care when it is done through technology. This could be because they lack understanding of how to do it, don’t have the right equipment, can’t afford data, or other reasons. We will ask patients about their experiences and work with them and staff to develop ideas about how to overcome barriers.

Workstream 3: We will take what we have learnt in Workstreams 1 and 2 to make suggestions to inform national stakeholders and to influence policymakers.

Patients and members of the public helped shape the research design. They continue to help guide our research by reading our reports, giving us their opinions and advising on how best to share our research so everyone can benefit from what we have learnt. Our governance panel is chaired by a member of the public.

## Background

### Remote general practice during the pandemic

The COVID-19 pandemic has been a crisis opportunity for digital innovation ^1^. In early 2020, remote—telephone, video and electronic—consultations were quickly introduced into UK general practice ^2, 3^. For various reasons, video was little used even at the height of the pandemic ^4, 5^. Practices introduced telephone and in some cases online consultations (patients complete a web template and receive email reply or call-back ^6^). Implementation challenges were common ^5, 7– 10^, and practices worked hard to retain a face-to-face service for vulnerable and complex patients ^11^. In July 2020, the UK’s Secretary of State for Health declared—prematurely as it turned out—that remote would be the default option for the indefinite future ^12^.

The shift to remote general practice was initially supported as a ‘heroic’ response to COVID-19 but later questioned as unsatisfactory and potentially unsafe ^13, 14^. Commentators raised concerns about access, continuity of care, diagnostic errors, loss of the ‘doorknob consultation’ (in which a patient raises a serious concern only as they are leaving ^15^), safeguarding challenges, and unsafe prescribing ^16– 19^. As one commentary put it, *“[we should] not assume that what has been necessary in a crisis represents what patients or clinicians want or need beyond*” (page 345) ^20^.

Whilst our own in-pandemic research found examples of high-quality remote care ^8– 10^, we affirmed these concerns and identified six new kinds of risk: a) practice organisation and set-up (digital inequities which restricted access, technologies that were unreliable and unfit for purpose, and reduced service efficiency); b) communication and the therapeutic relationship (a shift to more transactional consultations); c) quality of clinical care (including missed diagnoses, safeguarding challenges, over-investigation and over-treatment); d) increased burden on the patient (e.g. to self-examine and navigate between services); e) fewer opportunities for screening and managing the social determinants of health; and f) adverse impact on workforce (clinician and staff stress and compromised learning) ^21, 22^.

### Remote general practice before the pandemic

Until early 2020, remote general practice consultations had been technically possible but (with the exception of telephone triage ^23^) not widely used in UK ^24– 26^. Pre-pandemic research on telephone ^23, 27– 29^, video ^26, 30, 31^ and online consultations ^32– 35^ was typically couched in an efficiency narrative and dominated by randomised controlled trials or quasi-experimental designs in which success was measured in economic metrics such as consultation length, number of problems raised, number and type of follow-up encounters, and by ‘non-inferiority’ in clinical outcomes and patient and staff satisfaction ^23, 26, 27, 31, 36^.

Efficiency and satisfaction are important concerns, but this early literature focused more on remote *consultations* under controlled conditions than on the wider question of introducing remote care as a *service*. It showed, broadly speaking, that remote modalities were acceptable, safe and cost-effective in the circumstances studied. But this research rarely demonstrated the hoped-for improvements in service efficiency—indeed, they often showed that remote modalities *reduced* efficiency as a result of double-handling or more service contacts ^23, 27– 29, 32, 37, 38^. Some pre-pandemic studies had revealed remote-associated compromises to quality of care such as increased antibiotic prescribing ^39^. A sparse and somewhat speculative literature promotes remote services as a means of reducing greenhouse gas emissions (e.g. from traveling to appointments) ^40– 42^, though this literature rarely considers the unintended environmental effects if remote services over-diagnose, over-investigate, over-prescribe, over-refer or result in missed diagnoses and future emergency admissions.

The strengths and limitations of large-scale quantitative studies for evaluating remote service models were illustrated by a study of ‘telephone first’ (in which all patients first speak to a clinician and some are invited to attend in person) using mainly quantitative methods with a small qualitative component ^43^. Whilst, *on average*, telephone first led to an 8% increase in clinician workload with similar patient satisfaction and service usage to traditional models, there was huge diversity—with some practices reporting improved efficiency and access and others reporting the opposite. The authors commented that their methodology was not designed to explore how or why multiple interacting factors played out differently in different settings. Similarly, a rapid evidence synthesis of ‘digital first’ studies found that most had “very narrowly evaluate[d] the introduction or use of a class of technology (e.g. internet video consultation), rather than the integration of such technologies as part of a broader reorganisation or reimagining of services” (page 7), and that despite extensive primary research, “little evidence exists on outcomes related to quality of care, service delivery, benefits or harms for patients, or on financial costs/cost-effectiveness.” ^35^

With few exceptions, then, pre-pandemic studies comparing remote with conventional appointments lacked descriptive detail and nuance. A sparse literature of qualitative and mixed-method case studies had begun to document technical, logistical and regulatory hurdles to digital general practice ^25, 32, 35, 44– 46^. The pandemic provided impetus for widespread organisational change at pace and scale, supported by dedicated funding and relaxing of red tape ^1, 2, 5, 8– 10, 47^. But whilst these are excellent preconditions for innovation, *sustaining* such innovations long-term raises new challenges and is considerably more difficult ^9, 48^. 

### Digital inequity—a new component of inverse care

Digital inequity means unequal access to healthcare resulting from poor digital access, digital literacy or both ^49^. It tends to affect those with multiple other kinds of disadvantage such as poverty, low health literacy, poor housing, weak social networks, psychological stress (e.g. from fear of crime) and—for some—language and cultural discordance, which together may increase their vulnerability to illness, disease and disability ^50^. Tudor Hart’s inverse care law (people most in need of health care are least likely to seek it or receive it) reflects two mutually-reinforcing phenomena: worse health in deprived communities and also barriers to their access to healthcare ^51^; such inequities have worsened recently ^52, 53^. SARS-CoV-2 produced a *syndemic* as well as a pandemic – i.e. it exacerbated, and was exacerbated by, social and economic inequities ^54^.

The proportion of the public classed as “internet non-users” has fallen but there remain substantial inequities by social determinants such as geographical location, age, ethnicity and gender ^50, 55, 56^. The digital divide operates not just in terms of basic internet access but in terms of *how much* bandwidth, data bytes, connectivity, compatibility, confidence, skills, power (e.g. over who in the household has use of a computer or smartphone) people have, and the size and nature of the social networks they can draw on for assistance ^57^. Even basic technologies such as the telephone can exclude some individuals (e.g. hard of hearing, geographically off grid).

The NHS Widening Digital Participation programme 2017–20 aimed to ensure equity in access and care regardless of digital preferences ^58^. An independent evaluation ^59^ inspired various proposed solutions including raising awareness, digital skills training, digital champions, intergenerational mentoring, free public Wi-Fi, assistive technology and social prescribing ^60^. A qualitative systematic review recommended using diverse ways of raising awareness and inviting (e.g. online, paper, word of mouth); proactive outreach (e.g. working through agencies); partnering trusted professionals (e.g. GPs); and checking that digital interventions meet people’s needs ^61^. A paper on digital inclusion in the homeless talked of “assertive outreach” partnering with public and third-sector agencies ^62^.

These recommendations have influenced our study design. But we believe a ‘deficiency’ framing (patients depicted as lacking devices, data, connectivity, awareness, skills, confidence and support, all assumed rectifiable by interventions) overlooks the pervasive impact of multiple interacting social determinants ^50, 63^. We hypothesise that non-digital options, easily accessible in traditional ways, will be needed for some patients. Such options are often offered on an ad hoc basis at the discretion of individual staff rather than as agreed policy. Access arrangements for some groups (e.g. disability, pregnancy) are protected under the UK’s Equality Act (2010) which requires “reasonable adjustments”, but people who are—for example—just poor or with complex needs (such as drug or alcohol problems or victims of domestic violence) do not have the same level of legal protection.

### The need for detailed, in-depth case studies

In sum, whilst remote consultations have clinical potential in general practice, remote services are difficult to set up, technically challenging, may increase workload at a stressful time, and could worsen health inequities. Despite much research, remarkably little is known about the fine-grained detail of implementing and sustaining remote services in different general practice contexts. As Flyvbjerg has put it, *“a scientific discipline without a large number of thoroughly executed case studies is a discipline without systematic production of exemplars, and ... a discipline without exemplars is an ineffective one”* (page 219) ^64^. 

Mindful of this gap in the literature, we sought to study a small but diverse sample of cases in depth to produce rich explanations of complex phenomena and generate lessons from the similarities and contrasts between them.

## Methods

### Aims, objectives and research questions

**Figure 1.**
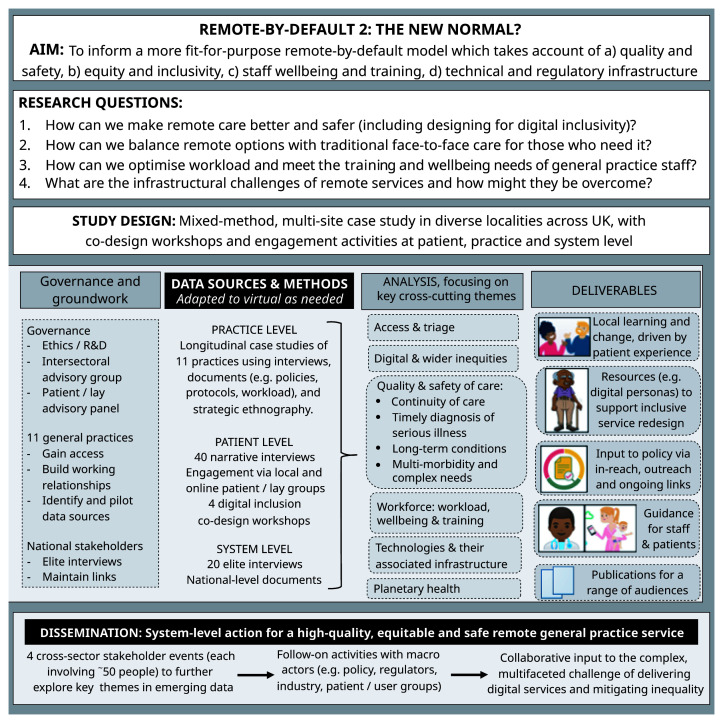
Study flowchart.

These are summarised in the flowchart in Figure 1.

### Study design

Mixed-methods multi-site case study with co-design and national stakeholder engagement. The study has three workstreams. Workstream 1 will use an embedded researcher-in-residence model to develop a multi-site longitudinal case study of general practices. Workstream 2 will capture patient experiences and use co-design with patients and staff to re-imagine service models and address digital inequities. Workstream 3 will engage national-level stakeholders and build networks for disseminating outputs.

### Workstream 1: Case studies in general practice

The goal is to produce detailed exemplars of complex change at practice level. We have recruited a sample of 11 general practices ( Table 1) for maximum variety in geographical location (six in England, two in Scotland and two in Wales), list size (from 2,800 to 33,500), practice demographics (extremely deprived to moderately affluent, skewed towards the former), ethos (varying, for example, in the value placed on own-doctor appointments), and digital maturity. 

**Table 1.** Participating practices.

Practice pseudonym	Location and rurality	List size	Practice demographics	Staffing	Digital maturity
Carleon	Wales; rural and remote	7,500	Farming community (Welsh-speakers, rural poverty); seasonal tourists	5 partners, few attached staff	Level 1 (traditional)
Camp St	England; urban (commuter town)	31,000	Stable population from wide range of ethnic and social backgrounds	20 doctors, many additional staff, teaching and training practice	Level 4 (digitally strategic)
Fernleigh	England; semi-rural (‘retirement’ village)	15,000	Mostly affluent and older white population; some rural poverty; one traveller site	7 partners, 40 staff including 7 salaried GPs, teaching and training practice	Level 3 (digitally curious), approaching Level 4
Newbrey	England; suburban (outskirts of university city)	21,000	Mixed population with young professionals and service-sector workers	5 GP partners, 9 salaried GPs, 2 nurse practitioners, 6 additional nurses	Between Level 2 (traditional with lone innovator), and Level 3
Ogden East	England; urban (deprived borough in small city)	8,000	Mostly white working-class with high rates of unemployment. Includes homeless hostel.	2 GP partners, 5 salaried GPs, 1 paramedic, 5 nurses. Offers drug and alcohol service.	Level 3 but very keen to avoid digital exclusion
Perrymore	England; urban but remote from tertiary care	33150	Mixed demographic across 7 sites, skewed to areas of high deprivation. Population mostly semi-skilled and unskilled; many refugees and asylum seekers.	9 GP partners and a managing partner, plus 17 GPs, 3 advanced nurse practitioners, 3 paramedics.	Level 3
River Rd	Scotland; inner-city (‘deep end’ practice)	5000	Mainly white working class but includes immigrants and displaced people.	4 GP partners, 1 nurse, plus linked district nurses and community staff.	Level 3
Rhian	Wales; urban (small town)	11,500	Mixed (former miners, retired people, young professionals). Long-established practice but premises viewed as old and unfit for purpose.	5 GP partners, 2 salaried GPs, 4 nurses, trainees and undergraduates. Several impending retirements.	Level 2, aspiring to Level 3 (was an early digital adopter but ‘fell behind’)
Range Park	Scotland; inner city (‘deep end’ practice)	2300	Very high levels of deprivation and low health literacy. Drug and alcohol problems.	3 GP partners plus various support staff.	Level 1 (“even phone connection can be poor”)
Towerhill	England; city-centre (central London borough)	16,000	Mixed (fairly affluent) population, many professionals, very ethnically diverse.	4 GP partners, 4 salaried GPs, many attached staff. Leads local GP federation.	Level 4, and in some respects Level 5 (system-oriented)
Westerly	England; inner city (deprived borough, now gentrifiying)	27,000	Ethnically and socially diverse; high turnover and many students.	6 GP partners, ~40 staff including 6 salaried GPs, 2 registrars, 7 nurses	Level 3

Our digital maturity scale ( Table 2) is simple and pragmatic. It draws on earlier work including a 136-item digital maturity self-assessment survey ^65^ which informed the NHS Five-year Forward View ^66^ but was abandoned soon after; a digital maturity matrix for electronic records ^67^; and the IDEAL framework for surgical innovations (typically technologies) ^68^. Our scale incorporates practices’ *readiness* (strategic alignment, leadership and resources), *capability* (remote services up and running) and *infrastructure* (the underpinning material, regulatory and human resources needed to accommodate new technologies and work routines). As Table 1 shows, two practices currently self-classify as ‘traditional’ (lowest level of digital maturity) and one is already ‘system-oriented’ (highest level). 

**Table 2.** Digital maturity scale for general practices. *Adapted from Greenhalgh et al. ^9^*.

Descriptor	How the practice currently supports remote consultations
LEVEL 1: TRADITIONAL (reactive)	Limited leadership or vision for developing remote services (perhaps for strategic reasons). Telephone is used for triage and call-backs. Little or no online access for patients; video and telehealth unavailable. Key infrastructure is probably absent. Digital inequities are addressed by focusing on face- to-face services.
LEVEL 2: TRADITIONAL WITH LONE INNOVATOR (ad hoc, demonstration)	The practice is traditional but one staff member is enthusiastic about remote care. They attempt to use novel technologies and engage others but have not yet succeeded in getting others to share the vision, influence practice strategy or change practice routines or policies. Infrastructure may be inadequate. Digital inclusion is not yet a priority issue.
LEVEL 3: DIGITALLY CURIOUS (experimenting)	The practice has a vision and plans for providing remote care. Traditional and new technologies are used creatively, and adjusted iteratively, to try to improve an aspect of care within the practice. Attempts are made to overcome digital inequities. Focus is on technical details and feasibility (i.e. making something work). Infrastructure is adequate but has some limitations.
LEVEL 4: DIGITALLY STRATEGIC (learning and improving)	The practice uses traditional and new technologies creatively and strategically, and evaluates benefits and disbenefits with the aim of improving care in all relevant areas, including efforts to meet the needs of digitally excluded groups. Digital capability is high (i.e. many services are successfully delivered remotely). Focus is on quality improvement and organisational learning. Work practices and routines are continuously adapted. Material and technical infrastructure is good as a result of strategic investment.
LEVEL 5: SYSTEM-ORIENTED (extending and spreading)	The practice has a clear vision and strategy for an effective, efficient, equitable remote service. Digital capability is high. Staff are actively involved in developing, evaluating and improving remote services both within and beyond the practice – e.g. through inter-organizational benchmarking, quality improvement collaboratives, locality-wide planning, research, or national guideline development.

Each practice has a researcher-in-residence, tasked with becoming familiar with its context and activity over two years (see Figure 1 for data sources). Beginning with informal interviews with a lead clinician or practice manager, they will arrange further interviews with practice staff and invite them to provide documents (e.g. practice leaflets, annual reports, audits). Case studies will be built iteratively and adaptively, depending on experiences and priorities salient locally. The researcher-in-residence will maintain a single point of contact for the practice, engage and work with their patient representatives, and keep the practice informed of activities (such as virtual workshops) and new resources (e.g. guidance) as these appear. Where appropriate and invited, researchers-in-residence will attend practice meetings (either face-to-face or via video link).

Staff interviews will combine a few basic semi-structured prompts (including “tell me about your job”, “what is your experience of remote access and remote consultations in the practice?”) with open-ended narrative probes (e.g. “can you tell me more about that?”, “what happened next and how did everyone react?” and “could you give me a story to illustrate that?”). Narrative interviews are conversational in format, seeking context and descriptive richness through examples; they are particularly useful in identifying emotive touchpoints and going beyond superficial description to capture meaning and significance ^69^.

Subject to pandemic restrictions, researchers-in-residence will undertake ethnography, focusing on what Star called the ‘ethnography of infrastructure’ ^70^. We will use strategic ethnography: a focused and historically-informed approach to data collection and analysis that considers the biography of artefacts in organisations—that is, how they emerged, their inter-relationships and inter-dependencies, and what they are becoming ^71^. Artefacts (most obviously, the hardware and software for phone, video and online consultations and triage, along with what some authors have called ‘in-between’ artefacts such as sticky notes, whiteboards and informal note systems ^72^) are considered as evolving components of a complex, dynamic system, with multi-sited ethnography providing “ *robust, contexted understandings of complex objects”* (page 527) ^71^.

Limited quantitative data (e.g. staffing levels, uptake data on different kinds of appointment) will be incorporated where relevant as part of an evolving longitudinal story of a changing practice in a changing context.

Research team members will meet every 2–4 weeks to compare and contrast across evolving cases, focusing on the key cross-cutting themes shown in Table 3 (which have emerged from the literature as well as from our early familiarisation interviews) and connect with other work packages.

**Table 3.** Cross-cutting themes to be explored across 11 general practices.

Theme	Rationale	Approach
Access and triage	‘Total triage’ (all initial contact to be by telephone and electronic form), introduced in March 2020 ^3^, has evolved in various forms since. In many practices, current systems are experienced as inefficient and hard to navigate ^5, 9^.	Using interviews, patient information resources and digital walk-throughs, we will map the patient pathway through the ‘digital front door’ and follow each practice’s efforts to improve accessibility and efficiency.
Digital and wider inequities	Quantitative studies have shown inequities by age, gender and ethnicity in remote consultation uptake ^74^, but those designs did not allow in-depth analysis of intersectionality—how different social determinants (e.g. being elderly *and* poor *and* chronically sick) combine and interact.	Using narrative interviews and ethnography, we will capture the complexities of disadvantage and exclusion for particular groups. Using co-design, we will develop digital personas to serve tools for re-imagining service provision.
Quality and safety of care	Remote care may compromise the therapeutic relationship and continuity of care ^19, 20^, lead to more transactional forms of clinical interaction ^22^, fewer ‘doorknob consultations’ ^15^, and delayed diagnosis of serious illness ^75, 76^; it may be unsuitable for those with complex needs ^20– 22^. Remote reviews may be convenient and safe for patients with stable long-term conditions ^77^.	Using staff and patient interviews, practice documents, ethnography, and video/audio of consultations (if feasible), we will explore how quality goals are achieved (or why they are not achieved) for different conditions and patient groups—including long-term conditions, multi-morbidity, early diagnosis of cancer, and vulnerable patients.
Workload, workforce and staff wellbeing	UK general practice is under system stress ^78^, with high and rising workload ^79^, task shifting from other sectors ^80^, a retention crisis ^81, 82^, and high levels of stress and burnout among clinicians, trainees and administrative staff ^83– 85^. Remote consulting is cognitively demanding ^86^ and may reduce opportunities for learning ^87^. Trainees report low confidence in assessing patients by telephone ^88^.	Workload and wellbeing will be a key focus of staff interviews and learning sets. Sub-studies will explore front-desk and back-office work routines ethnographically; study experiences of under-researched lower-grade staff (receptionists, administrators, cleaners) and those of trainers and trainees.
Technologies and their associated infrastructure	There is a longstanding policy push to strengthen NHS digital infrastructure ^89– 92^. Some technologies developed during the pandemic bypassed regulatory approvals ^1, 93^. In some cases, products approved at speed at the height of the pandemic subsequently proved unfit for purpose. The procurement process for new technologies was sometimes poorly aligned with business cycles.	Using interviews, ethnography, digital walk-throughs and analysis of relevant national and local IT policies, we will study both the novel technologies and the material and digital infrastructure, human resources, technical expertise and business decisions needed to support and troubleshoot technology adoption and use.
Patient input to practice improvement	Drawing on patients’ experience (and trying to improve it) is a well-established method for service improvement ^70^. with strong theoretical grounding in phenomenology ^94^. Many but not all general practices have established patient participation groups.	Researchers-in-residence will work flexibly with each practice and (where established) local patient involvement groups, incorporating additional insights from patient interviews, our patient learning set, and lay input to stakeholder workshops.
Planetary health	Travel to healthcare appointments (e.g. by car) generates greenhouse gases ^95, 96^. Remote service provision could potentially reduce this, though carbon savings in primary care may be modest, and could be achieved at the expense of waste (e.g. over-diagnosis, over-treatment or over-referral). Local savings (of various kinds) may come at the expense of ‘hidden’ environmental waste (e.g. data warehousing).	We will calculate the carbon footprint of a sample of consultations and linked patient pathways in some practices. We will explore critical events with potential for adverse carbon impact (e.g. when patients are sent for tests rather than being examined face to face). We will explore the extent to which environmental sustainability is (or could be) built into practice business cases.

We will apply the principles of action research ^73^ (taking an iterative and collaborative approach *with* practice members; establishing locally-appropriate ways to rapidly evaluate and feed into learning; and seeking participation and buy-in from staff and patients) to support each practice in its efforts to learn and develop around four key goals: a) optimising quality and safety of care; b) ensuring digital inclusion and providing equitable alternatives for the digitally excluded; c) addressing staff wellbeing and training; and d) overcoming infrastructural hurdles (both technical and regulatory). Action research has two goals: supporting local change (hence, benefits for local patients and staff) and producing generalisable learning (through the generation of rich, case-based understanding which supports theorising).

In a sub-sample of three practices (Camp St, Fernleigh and Towerhill), we will undertake detailed ethnography of front-desk and back-office work on tasks such as appointment booking, call handling and triage, with a view to teasing out key workplace routines and exploring their interdependencies and implications. We will supplement this with analysis of routine practice data on use of different consulting modalities over 24 months to appreciate service use and changes over time. In another sub-sample (yet to be identified), we will calculate the carbon footprint of a sample of consultations and linked patient pathways (e.g. referrals, investigations), mindful that these pathways may differ in remote versus face-to-face consultations. We will explore how practices are incorporating (or why they are not incorporating) sustainability considerations into their strategies and business plans, and in decisions about how and why different consultation modalities are (or aren’t) being used.

Mindful of the established value of inter-organisational networking and support in complex change ^48^, we will offer practices a series of webinars, link them to a range of resources (e.g. clinical standards and guidance, patient resources) as we develop these, and set up an e-mail discussion list for key practice contacts.

### Workstream 2: The patient perspective and co-design

The goal is to support inclusion of patient and carer perspectives in the design and redesign of remote services. Sampling 40 participants, we will seek to maximise diversity in age, socio-demographic background, ethnicity, housing status (e.g. homeless or ‘sofa-surfing’, privately rented, owner-occupied), digital literacy, confidence, and nature of illness or condition(s). We will ask practice staff to nominate patients and work with practice patient involvement groups and patient advocacy groups external to the practice (who often have strong online presence but also well-established ways of reaching less digitally confident members). We will also snowball from participants, asking them to nominate and ‘buddy’ a friend or relative (e.g. a young second- or third-generation immigrant from a minority ethnic group may be able to connect us to a grandparent who speaks limited English and limited digital experience or access).

Working both locally and at the level of national advocacy groups will allow us to include a perspective on what it is like being cared for in participating practices as well as a more generic patient voice for certain conditions. We will ask practices, patient groups and snowball contacts to identify people who they think may have found it challenging to consult remotely, as well as those who are keen and confident to help advise and support others. Carers of people unable to give a full account of their own experience (e.g. cognitive impairment) will also be included in the sample. We will note advice given by one of our patient advisers that people may be very digitally literate on certain platforms (e.g. Facebook) but less so on others (e.g. online consultation forms).

Potential patient participants will first be approached by someone outside the research team (practice staff or fellow patient). Participation is voluntary; they can withdraw at any time and personal details will be anonymised. They may choose video, telephone or face-to-face format (e.g. homeless people will be interviewed at lunch clubs in a private space). Interviews will combine basic semi-structured prompts (e.g. “how long have you been a patient in the practice?”, “what illnesses or conditions do you receive care for?”, “what is your experience of booking and having consultations remotely?”) with and narrative probes (conversational, seeking examples and depth for whatever the patient chooses to talk about).

Interviews aim to capture the patient and carer experience of remote services across four key quality and safety areas (long term condition monitoring, getting an appointment with own clinician and maintaining continuity of a therapeutic relationship over time, presenting with symptoms that could indicate early cancer, and care for multimorbidity and other complex needs). Findings will be fed into digital inclusion co-design workshops—two with patients and carers, and two with practice staff (along with patient representatives), described in Box 1.

**Box 1. Inclusive digital transformation**

A non-profit digital co-design agency ( *Thrive by Design*), with an interest in digital inclusion ^59^ will use a validated action research methodology for supporting inclusive digital transformation.

*Thrive by Design* will begin by working with three participating practices to run digital inclusion co-design workshops using the guiding question: *“How can we best provide safe and effective care through remote consultations, and what measures do we need to put in place for people for whom standard remote consultations are unsuitable or unacceptable?”.*

One output of such workshops will be a range of *digital inclusion personas.* These are fictional characters who encompass features we need to think about when selecting technologies and designing and embedding technology-aided services (e.g. Fred is a 35-yr-old heroin addict living in cardboard city who gets his methadone from an NHS general practice) ^97, 98^.

Working across the three initial practices, the first co-design workshop will be held with patients and carers, either virtually or in-person. People less comfortable with the virtual format will be supported to contribute using telephone in an asynchronous format (i.e. building a picture over several days/weeks). The personas and wider insights generated by these patient and carer workshops will be used to inform and enliven two additional workshops for practice staff (including clinicians, managers, administrators and patient and lay representatives across the three practices). Preparatory briefing materials will be sent out beforehand. Participants will work partly in virtual breakout rooms to think creatively about meeting the needs of the different digital inclusion personas.

The outputs of these workshops are unlikely to be simple or universal solutions. We anticipate they will generate ideas for how (and for whom) to deploy existing remote technologies, additional off-the-shelf or bespoke products which could enhance provision, and novel service models. The format will be extended to other participating practices as the study unfolds.

### Workstream 3: National stakeholder engagement and dissemination work

We use ‘élite’ national stakeholder interviews for two purposes: to gather data on the macro-level policy, infrastructural and regulatory context including public-private partnerships, financing and reimbursement and so on; and to build strategic links for future dissemination. To sample participants, we will draw on our diverse external advisory group and our established links with Digital First Primary Care Team at NHS England and the TEC (Technology Enabled Care) teams in Scottish and Welsh governments, NHS leaders (including clinical directors, chief clinical information officers and informal digital champions), those in industry (both large technology providers and start-ups, many of whom developed new products during the pandemic and made these available free or at low cost to the NHS), professional bodies (including Royal Colleges) and advisors (e.g. defence societies), regulators (such as National Institute for Health and Clinical Excellence, General Medical Council and Medicines and Healthcare Devices Regulatory Agency), and third-sector groups including patient advocacy groups.

We use a combination of about 20 initial quick, informal interviews (often very helpful to glean over-arching themes and issues) and at least 20 more formal semi-structured and narrative interviews. The former will not be audiotaped (and hence may provide opportunity for candid insights) but we will take contemporaneous notes. The latter will be recorded and professionally transcribed. We will invite elite interviewees to recommend key documents that are guiding their field (e.g. policies, regulation, guidance) and ‘follow the trail’ of these documents. Where appropriate, we will snowball (i.e. ask interviewees to nominate another senior stakeholder and introduce us by email).

As our study progresses, we will hold four cross-sector stakeholder events using a method developed by our partner the Nuffield Trust. These will begin virtually but may revert to a face-to-face format. We anticipate that workshops will cover the priority topics listed in Table 3.

For each workshop, we will identify a wide mix of stakeholders (including patient groups) whose perspectives are relevant to the chosen theme, make personal contact to invite and engage them, and prepare and circulate a preliminary resource pack (with key materials such as an agenda and objectives, a lay summary of our research, digital inclusion personas, an anonymised and fictionalised significant event). The workshop will begin with a short plenary before participants discuss topics in breakout groups. A final plenary will bring groups together to report back, continue discussion and identify specific steps which need to be taken. 

Follow-up activities will include meetings with particular stakeholders, convening smaller task and finish groups (e.g. to prepare a policy briefing), or planning a new stream of research. 

### Data management and analysis

All formal interviews and ethnographic field notes will be transcribed, de-identified and stored on an encrypted server at the University of Oxford, which will also be used to store research diary notes, key emails and correspondence, facilitator notes, chat comments and reports from online workshops, and public-domain local and national documents. We will use NVIVO, which allows for easy storage, indexing, coding and cross-linking. We will code data thematically to gain familiarity and also analyse relevant segments narratively by asking questions about characters, emplotment and emotional touchpoints ^69^.

To initiate and build on practice-based case studies and cross-case comparisons, will use hermeneutic methods, in particular the constant comparative method described by Glaser ^99, 100^, in which each new data item is added to a progressively richer picture of the whole. For each practice case study, we will combine the various data sources (interviews, ethnographic observations, documents, quantitative data) to build a rich narrative of the local emergence, current use and intended evolution (or replacement) of these artefacts over both short and long temporal scales, attending in particular (but not exclusively) to the priority themes in Table 3.

Each researcher-in-residence has drawn together early interviews and data sources to prepare an initial practice familiarisation document. These summarise the background and context for the 11 participating practices and the issues and challenges each currently faces. These interim summaries are being compared and contrasted in cross-case review meetings, leading to refinement of the cross-case themes. Narrative methods will be crucial for drawing out understanding of micro-level causal pathways which explain (e.g.) why something that ‘succeeded’ in one setting ‘failed’ in another. Narrative richness will also allow us to identify and test demi-regularities (things that tend to be the case in particular circumstances) and candidate explanatory theories. Key to cross-case analysis is reflection and discussion among the embedded researchers, and also among patient representatives in the different practice settings. As the study progresses, we will add detail to individual practice summaries and the over-arching summary of cross-case themes. We will seek disconfirming data (qualitative or quantitative data which would lead us to question our current understanding) and use these to amend or refine our understanding.

The same approach will be taken for patient interviews, material from patient workshops, and national stakeholder interviews. In each case, an initial summary document will be prepared through thematic and narrative analysis of the first few interviews, and this summary will be progressively refined as each additional interview is added ^100^. We will use member checking to clarify accuracy and interpretation of interview data.

### Linked PhD projects

A linked PhD by EL (funded by the NIHR School of Primary Care Research) will track consulting activity for 30 patients with complex needs ^101^ (10 in each of three practices) over a two-year period; detailed methodology for this study is under development. Two additional PhDs are based in primary care settings outside our sample of 11 practices so as not to overload them. FD (funded by NIHR School of Primary Care Research) will explore the experiences of under-researched lower-grade staff such as receptionists, administrators and cleaners as practices move towards remote care as business as usual. LH (funded by THIS Institute) will study the patient experience of accessing remote care in patients with multiple disadvantage (elderly, lower socio-economic groups, limited English speakers). AB's PhD (funded by Rhodes Trust) is exploring aspects of sustainability and carbon-reduction policies relating to pharmaceutical supply and provision, including how decisions about sustainable prescribing are influenced by the shift to remote assessment and monitoring. 

### Patient and public involvement

There is extensive lay representation on the external advisory group (see below) including a lay chair (AAN, co-author). We have strong links to local patient involvement groups in participating practices where these exist. AAN has established a patient / lay involvement virtual group with representation across participating practices and an arrangement where those in the group commit to buddying others who are not online (or less confident online). Patients and lay people have been formatively and iteratively involved in designing the study; their input has been crucial to shaping the original bid (especially the kinds of remote consultations they are most concerned about) and in responding to changes as the study unfolds. All inter-sectoral workshops include patient and lay participants.

## Ethics and dissemination

### Governance

The study has an independently chaired external advisory group with diverse representation from policy, clinical care, the commercial sector, people with lived experience, and members of patient advocacy groups and regulatory bodies. It receives a three-monthly written progress report before an advisory group meeting with the research team. The advisory group’s comments are summarised in writing and taken forward by the core research team.

### Ethical approval and consent

Approval has been granted from East Midlands—Leicester South Research Ethics Committee and UK Health Research Authority (September 2021, 21/EM/0170) and subsequent amendments. All patients and staff interviewed gave written informed consent in accordance with our ethics protocol. The ethics committee have approved easy-read versions of the information sheets and consent forms for low-literacy participants.

### Study status

We have collected and analysed baseline data on all practices, which we have presented in a separate paper ^102^. Selected additional data on this ongoing, mainly qualitative study will be made available to researchers on reasonable request to the lead author (TG). We anticipate that data collection for this study will be complete by August 2023 and analysis complete by end November 2023.

### Other planned outputs

Future academic outputs will include empirical studies describing anonymised case studies and cross-case analyses, and—we anticipate—demonstrating the links between digital inequities and the wider social determinants of health. We also plan theoretical and methodological outputs—covering (for example) the challenges and contribution of small-scale in-depth case studies for addressing complex change in the digital world and understanding causality. We will use the co-production aspects of this study design to generate guidance and tools (including digital personas) on developing effective remote services and also patient-facing resources (including an animation) on securing and navigating one’s digital appointment.

## Conclusion

This study does not promise easy or universal answers to the question of how remote modalities can be maintained as part of a mixed-modality general practice service, nor how they impact on digital equality. However, our focus on depth and detail in a small sample of practices with different histories, geographies and current challenges will illuminate the complexity of the “new normal” and provide the case exemplars which are crucial to understanding social phenomena and supporting service improvement.

## Reporting guidelines

We have followed published guidance for case study research ^64^. Formal, structured protocols akin to CONSORT for randomised controlled trials do not exist for this kind of research.

## Data availability

### Underlying data

No underlying data are associated with this article.

## References

[1] Gkeredakis M Lifshitz-Assaf H Barrett M : Crisis as opportunity, disruption and exposure: Exploring emergent responses to crisis through digital technology. *Information and Organization.* 2021;31(1):100344. 10.1016/j.infoandorg.2021.100344

[2] Joy M McGagh D Jones N : Reorganisation of primary care for older adults during COVID-19: a cross-sectional database study in the UK. *Br J Gen Pract.* 2020;70(697):e540–e47. 10.3399/bjgp20X710933 32661009 PMC7363277

[3] NHS England: Advice on how to establish a remote ‘total triage’ model in general practice using online consultations. London: NHS England.2020. Reference Source

[4] Greenhalgh T Ladds E Hughes G : Why do GPs rarely do video consultations? qualitative study in UK general practice. *Br J Gen Pract.* 2022;72(718):e351–e360. 10.3399/BJGP.2021.0658 35256385 PMC8936181

[5] Murphy M Scott LJ Salisbury C : Implementation of remote consulting in UK primary care following the COVID-19 pandemic: a mixed-methods longitudinal study. *Br J Gen Pract.* 2021;71(704):e166–e77. 10.3399/BJGP.2020.0948 33558332 PMC7909923

[6] Bakhai M Atherton H : How to conduct written online consultations with patients in primary care. *BMJ.* 2021;372:n264. 10.1136/bmj.n264 33627324

[7] Tuijt R Rait G Frost R : Remote primary care consultations for people living with dementia during the COVID-19 pandemic: experiences of people living with dementia and their carers. *Br J Gen Pract.* 2021;71(709):574–82. 10.3399/BJGP.2020.1094 33630749 PMC8136581

[8] Wherton J Greenhalgh T Shaw SE : Expanding Video Consultation Services at Pace and Scale in Scotland During the COVID-19 Pandemic: National Mixed Methods Case Study. *J Med Internet Res.* 2021;23(10):e31374. 10.2196/31374 34516389 PMC8500351

[9] Greenhalgh T Rosen R Shaw SE : Planning and Evaluating Remote Consultation Services: A New Conceptual Framework Incorporating Complexity and Practical Ethics. *Front Digit Health.* 2021;3:726095. 10.3389/fdgth.2021.726095 34713199 PMC8521880

[10] Shaw SE Hughes G Wherton J : Achieving Spread, Scale Up and Sustainability of Video Consulting Services During the COVID-19 Pandemic? Findings From a Comparative Case Study of Policy Implementation in England, Wales, Scotland and Northern Ireland. *Front Digit Health.* 2021;3(194):754319. 10.3389/fdgth.2021.754319 34988546 PMC8720935

[11] Turner A Scott A Horwood J : Maintaining face-to-face contact during the COVID-19 pandemic: a longitudinal qualitative investigation in UK primary care. *BJGP Open.* 2021;5(5):BJGPO.2021.0036. 10.3399/BJGPO.2021.0036 34257067 PMC8596308

[12] Hancock M : The Future of Healthcare.(speech, 30th July). London: Gov.uk.2020; Accessed 12th November 2021. Reference Source

[13] Mroz G Papoutsi C Greenhalgh T : ‘From disaster, miracles are wrought’: a narrative analysis of UK media depictions of remote GP consulting in the COVID-19 pandemic using Burke’s pentad. *Med Humanit.* 2021;47(3):292–301. 10.1136/medhum-2020-012111 33782180 PMC8008912

[14] Mroz G Papoutsi C Rushforth A : Changing media depictions of remote consulting in COVID-19: analysis of UK newspapers. *Br J Gen Pract.* 2020;71(702):e1–e9. 10.3399/BJGP.2020.0967 33318086 PMC7759365

[15] Nielsen SB : Patient initiated presentations of additional concerns. *Discourse Studies.* 2012;14(5):549–65. 10.1177/1461445612454081

[16] Khan N Jones D Grice A : A brave new world: the new normal for general practice after the COVID-19 pandemic. *BJGP Open.* 2020;4(3):bjgpopen20X101103. 10.3399/bjgpopen20X101103 32487520 PMC7465568

[17] NIHR Applied Research Collaboration West: Collecting rapid COVID-19 intelligence to improve primary care response. Bristol: University of Bristol2020.

[18] Imlach F McKinlay E Middleton L : Telehealth Consultations in General Practice During a Pandemic Lockdown: Survey and Interviews on Patient Experiences and Preferences. *BMC Fam Pract.* 2020;21(1):269. 10.1186/s12875-020-01336-1 33308161 PMC7733693

[19] Gray DP Freeman G Johns C : Covid 19: a fork in the road for general practice. *BMJ.* 2020;370:m3709. 10.1136/bmj.m3709 32988832

[20] Swinglehurst D Dowrick C Heath I : ‘Bad old habits’ … and what really matters. *Br J Gen Pract.* 2020;70(699):485–86. 10.3399/bjgp20X712745 33004362 PMC7518894

[21] Rosen R Wieringa S Greenhalgh T : Clinical risk in remote consultations: findings from in-pandemic qualitative case studies. *Brit J Gen Pract.* 2021; in press.

[22] Wieringa S Neves AL Rushforth A : Safety implications of remote assessments for suspected COVID-19: qualitative study in UK primary care. *BMJ Qual Saf.* 2022;bmjqs-2021-013305. 10.1136/bmjqs-2021-013305 35260414 PMC8927927

[23] Campbell JL Fletcher E Britten N : Telephone triage for management of same-day consultation requests in general practice (the ESTEEM trial): a cluster-randomised controlled trial and cost–consequence analysis. *Lancet.* 2014;384(9957):1859–68. 10.1016/S0140-6736(14)61058-8 25098487

[24] Brant H Atherton H Ziebland S : Using alternatives to face-to-face consultations: a survey of prevalence and attitudes in general practice. *Br J Gen Pract.* 2016;66(648):e460–e66. 10.3399/bjgp16X685597 27215571 PMC4917048

[25] Atherton H Brant H Ziebland S : Alternatives to the face-to-face consultation in general practice: focused ethnographic case study. *Br J Gen Pract.* 2018;68(669):e293–e300. 10.3399/bjgp18X694853 29378697 PMC5863684

[26] Hammersley V Donaghy E Parker R : Comparing the content and quality of video, telephone, and face-to-face consultations: a non-randomised, quasi-experimental, exploratory study in UK primary care. *Br J Gen Pract.* 2019;69(686):e595–e604. 10.3399/bjgp19X704573 31262846 PMC6607843

[27] Downes MJ Mervin MC Byrnes JM : Telephone consultations for general practice: a systematic review. *Syst Rev.* 2017;6(1):128. 10.1186/s13643-017-0529-0 28673333 PMC5496327

[28] Newbould J Ball S Abel G : A ‘telephone first’ approach to demand management in English general practice: a multimethod evaluation. *Health Serv Del Res.* 2019;7(17):17. 10.3310/hsdr07170 31063292

[29] Thompson-Coon J Abdul-Rahman AK Whear R : Telephone consultations in place of face to face out-patient consultations for patients discharged from hospital following surgery: a systematic review. *BMC Health Serv Res.* 2013;13(1):128. 10.1186/1472-6963-13-128 23561005 PMC3626714

[30] Thiyagarajan A Grant C Griffiths F : Exploring patients' and clinicians' experiences of video consultations in primary care: a systematic scoping review. *BJGP open.* 2020;4(1):bjgpopen20X101020. 10.3399/bjgpopen20X101020 32184212 PMC7330183

[31] Donaghy E Atherton H Hammersley V : Acceptability, benefits, and challenges of video consulting: a qualitative study in primary care. *Br J Gen Pract.* 2019;69(686):e586–e594. 10.3399/bjgp19X704141 31160368 PMC6617540

[32] Farr M Banks J Edwards HB : Implementing online consultations in primary care: a mixed-method evaluation extending normalisation process theory through service co-production. *BMJ Open.* 2018;8(3):e019966. 10.1136/bmjopen-2017-019966 29555817 PMC5875620

[33] Baines R Tredinnick-Rowe J Jones R : Barriers and enablers in implementing electronic consultations in primary care: scoping review. *J Med Internet Res.* 2020;22(11):e19375. 10.2196/19375 33035177 PMC7674136

[34] Chambers D Cantrell AJ Johnson M : Digital and online symptom checkers and health assessment/triage services for urgent health problems: systematic review. *BMJ Open.* 2019;9(8):e027743. 10.1136/bmjopen-2018-027743 31375610 PMC6688675

[35] Rodgers M Raine GA Thomas S : Informing NHS policy in 'digital-first primary care': a rapid evidence synthesis. *Health Serv Deliv Res.* 2019;7(41):1–154. 10.3310/hsdr07410 31869020

[36] Chongmelaxme B Lee S Dhippayom T : The Effects of Telemedicine on Asthma Control and Patients' Quality of Life in Adults: A Systematic Review and Meta-analysis. *J Allergy Clin Immunol Pract.* 2019;7(1):199–216.e11. 10.1016/j.jaip.2018.07.015 30055283

[37] Salisbury C Murphy M Duncan P : The impact of digital-first consultations on workload in general practice: modeling study. *J Med Internet Res.* 2020;22(6):e18203. 10.2196/18203 32543441 PMC7327596

[38] Turnbull J Pope C Rowsell A : The work, workforce, technology and organisational implications of the ‘ 111’ single point of access telephone number for urgent (non-emergency) care: a mixed-methods case study. *Health Serv Deliv Res.* 2014;2(3). 10.3310/hsdr02030 27466643

[39] Han SM Greenfield G Majeed A : Impact of remote consultations on antibiotic prescribing in primary health care: systematic review. *J Med Internet Res.* 2020;22(11):e23482. 10.2196/23482 33031045 PMC7655728

[40] Holmner Å Ebi KL Lazuardi L : Carbon footprint of telemedicine solutions-unexplored opportunity for reducing carbon emissions in the health sector. *PLoS One.* 2014;9(9):e105040. 10.1371/journal.pone.0105040 25188322 PMC4154849

[41] Oliveira TC Barlow J Gonçalves L : Teleconsultations reduce greenhouse gas emissions. *J Health Serv Res Policy.* 2013;18(4):209–14. 10.1177/1355819613492717 23945677

[42] Wootton R Tait A Croft A : Environmental aspects of health care in the Grampian NHS region and the place of telehealth. *J Telemed Telecare.* 2010;16(4):215–20. 10.1258/jtt.2010.004015 20511579 PMC3104823

[43] Newbould J Exley J Ball S : GPs’ and practice staff’s views of a telephone first approach to demand management: a qualitative study in primary care. *Br J Gen Pract.* 2019;69(682):e321–e328. 10.3399/bjgp19X702401 31015225 PMC6478459

[44] Odendaal WA Watkins JA Leon N : Health workers’ perceptions and experiences of using mHealth technologies to deliver primary healthcare services: a qualitative evidence synthesis. *Cochrane Database Syst Rev.* 2020;3(3):CD011942. 10.1002/14651858.CD011942.pub2 32216074 PMC7098082

[45] Wherton J Shaw S Papoutsi C : Guidance on the introduction and use of video consultations during COVID-19: important lessons from qualitative research. *BMJ Leader.* 2020;4(3):120–23. 10.1136/leader-2020-000262

[46] Banks J Farr M Salisbury C : Use of an electronic consultation system in primary care: a qualitative interview study. *Br J Gen Pract.* 2018;68(666):e1–e8. 10.3399/bjgp17X693509 29109115 PMC5737315

[47] Marshall M Howe A Howsam G : COVID-19: a danger and an opportunity for the future of general practice. *Br J Gen Pract.* 2020;70(695):270–71. 10.3399/bjgp20X709937 32393503 PMC7219629

[48] Greenhalgh T Robert G Macfarlane F : Diffusion of innovations in service organizations: systematic review and recommendations. *Milbank Q.* 2004;82(4):581–629. 10.1111/j.0887-378X.2004.00325.x 15595944 PMC2690184

[49] Veinot TC Mitchell H Ancker JS : Good intentions are not enough: how informatics interventions can worsen inequality. *J Am Med Inform Assoc.* 2018;25(8):1080–88. 10.1093/jamia/ocy052 29788380 PMC7646885

[50] Zheng Y Walsham G : Inequality of what? An intersectional approach to digital inequality under Covid-19. *Information and Organization.* 2021;31(1):100341. 10.1016/j.infoandorg.2021.100341

[51] Hart JT : The inverse care law. *Lancet.* 1971;1(7696):405–12. 10.1016/S0140-6736(71)92410-X 4100731

[52] Marmot M : An inverse care law for our time. *BMJ.* 2018;362:k3216. 10.1136/bmj.k3216 30065009

[53] Mercer SW Guthrie B Furler J : Multimorbidity and the inverse care law in primary care. *BMJ.* 2012;344:e4152. 10.1136/bmj.e4152 22718915

[54] Bambra C Riordan R Ford J : The COVID-19 pandemic and health inequalities. *J Epidemiol Community Health.* 2020;74(11):964–968. 10.1136/jech-2020-214401 32535550 PMC7298201

[55] Office of National Statistics: Exploring the UK’s digital divide.London: ONS.2019; Accesed 12th November 2021. Reference Source

[56] Elahi F : Digital Inclusion: Bridging Divides.Windsor: Cumberland Lodge. Reference Source

[57] Hilbert M : The bad news is that the digital access divide is here to stay: Domestically installed bandwidths among 172 countries for 1986– 2014. *Telecomm Policy.* 2016;40(6):567–81. 10.1016/j.telpol.2016.01.006

[58] NHS England: Implementing phase 3 of the NHS response to the COVID-19 pandemic.London: NHS England.2020. Reference Source

[59] Stone E Nuckley P Shapiro R : Digital inclusion in health and care: Lessons learned from the NHS Widening Digital Participation Programme.Leeds: Good Things Foundation.2020; Accessed 12th November 2021. Reference Source

[60] NHS Digital: How we can support digital inclusion. London: NHS Digital.2020; Accessed 12th November 2021. Reference Source

[61] O’Connor S Hanlon P O’Donnell CA : Understanding factors affecting patient and public engagement and recruitment to digital health interventions: a systematic review of qualitative studies. * BMC Med Inform Decis Mak.* 2016;16(1):120. 10.1186/S12911-016-0359-3 27630020 PMC5024516

[62] Williams H Whelan A : An investigation into access to digital inclusion for healthcare for the homeless population. Hastings: Seaview.2017; Accessed 7th March 2021. Reference Source

[63] Marmot M Allen J Goldblatt P : Build back fairer: the COVID-19 Marmot review. The pandemic, socioeconomic and health inequalities in England. *London Inst Heal Equity.* 2020. Reference Source

[64] Flyberrg B : Five misunderstandings about case-study research. *Qual Inq.* 2006;12(2):219–45. 10.1177/1077800405284363

[65] Johnston DS : Digital maturity: are we ready to use technology in the NHS? *Future Healthc J.* 2017;4(3):189–192. 10.7861/futurehosp.4-3-189 31098469 PMC6502583

[66] NHS England: The forward view into action: planning for 2015/16. London: NHS England London: NHS England.2014. Reference Source

[67] Flott K Callahan R Darzi A : A patient-centered framework for evaluating digital maturity of health services: a systematic review. *J Med Int Res.* 2016;18(4):e75. 10.2196/jmir.5047 27080852 PMC4850277

[68] McCulloch P Altman DG Campbell WB : No surgical innovation without evaluation: the IDEAL recommendations. *Lancet.* 2009;374(9695):1105–12. 10.1016/S0140-6736(09)61116-8 19782876

[69] Bate P Robert G : Experience-based design: from redesigning the system around the patient to co-designing services with the patient. *Qual Saf Health Care.* 2006;15(5):307–10. 10.1136/qshc.2005.016527 17074863 PMC2565809

[70] Star SL : The ethnography of infrastructure. *Am Behav Sci.* 1999;43(3):377–91. 10.1177/00027649921955326

[71] Pollock N Williams R : E-infrastructures: How do we know and understand them? Strategic ethnography and the biography of artefacts. *Comput Support Coop Work (CSCW).* 2010;19(6):521–56. 10.1007/s10606-010-9129-4

[72] Pinder R : Betwixt and between: part-time GPs and the flexible working question. In: Malin N, ed. *Professionalism, Boundaries and the Workplace*. London: Routledge,2000.

[73] Koshy E Koshy V Waterman H : Action research in healthcare.Sage,2010. Reference Source

[74] Parker RF Figures EL Paddison CA : Inequalities in general practice remote consultations: a systematic review. *BJGP Open.* 2021;5(3):BJGPO.2021.0040. 10.3399/BJGPO.2021.0040 33712502 PMC8278507

[75] Sud A Torr B Jones ME : Effect of delays in the 2-week-wait cancer referral pathway during the COVID-19 pandemic on cancer survival in the UK: a modelling study. *Lancet Oncol.* 2020;21(8):1035–1044. 10.1016/S1470-2045(20)30392-2 32702311 PMC7116538

[76] Hamilton W : Cancer diagnostic delay in the COVID-19 era: what happens next? *Lancet Oncol.* 2020;21(8):1000–1002. 10.1016/S1470-2045(20)30391-0 32702312 PMC7834491

[77] Kelley L Phung M Stamenova V : Exploring how virtual primary care visits affect patient burden of treatment. *Int J Med Inform.* 2020;141:104228. 10.1016/j.ijmedinf.2020.104228 32683311

[78] Dominic C Gopal DP Sidhu A : ‘It’s like juggling fire daily’: Well-being, workload and burnout in the British NHS - A survey of 721 physicians. *Work.* 2021;70(2):395–403. 10.3233/WOR-205337 34633337

[79] Hobbs FDR Bankhead C Mukhtar T : Clinical workload in UK primary care: a retrospective analysis of 100 million consultations in England, 2007–14. *Lancet.* 2016;387(10035):2323–30. 10.1016/S0140-6736(16)00620-6 27059888 PMC4899422

[80] Speakman EM Jarvis H Whiteley D : Opportunities and risks within the expanding role of general practice. *Br J Gen Pract.* 2021;71(709):344–345. 10.3399/bjgp21X716489 34326075 PMC8312678

[81] Owen K Hopkins T Shortland T : GP retention in the UK: a worsening crisis. Findings from a cross-sectional survey. *BMJ Open.* 2019;9(2):e026048. 10.1136/bmjopen-2018-026048 30814114 PMC6398901

[82] Bostock N : Future of general practice at risk as one in six GPs to quit or retire early after COVID-19. GP Online Magazine,2020. Reference Source

[83] Bugaj T Valentini J Miksch A : Work strain and burnout risk in postgraduate trainees in general practice: an overview. *Postgrad Med.* 2020;132(1):7–16. 10.1080/00325481.2019.1675361 31570072

[84] Vera San Juan N Aceituno D Djellouli N : Mental health and well-being of healthcare workers during the COVID-19 pandemic in the UK: contrasting guidelines with experiences in practice. *BJPsych Open.* 2020;7(1):e15. 10.1192/bjo.2020.148 33298229 PMC7844154

[85] Dawnay G : Is this really doctoring? *Br J Gen Pract.* 2020;70(698):455. 10.3399/bjgp20X712445 32855139 PMC7449438

[86] Ambrose L : Remote consulting: recognising the cognitive load. *Br J Gen Pract.* 2020;70(695):295. 10.3399/bjgp20X710213 32467208 PMC7241908

[87] Neve G Fyfe M Hayhoe B : Digital health in primary care: risks and recommendations. *Br J Gen Pract.* 2020;70(701):609–10. 10.3399/bjgp20X713837 33243917 PMC7707039

[88] Chaudhry U Ibison J Harris T : Experiences of GP trainees in undertaking telephone consultations: a mixed-methods study. *BJGP Open.* 2020;4(1):bjgpopen20X101008. 10.3399/bjgpopen20X101008 32019774 PMC7330189

[89] NHS England: Five Year Forward View.London: NHS England,2014. Reference Source

[90] NHS England: NHS Long Term Plan.London: NHS England,2019. Reference Source

[91] Monitor Deloitte: Digital Health in the UK: An industry study for the Office of Life Sciences.London: Deloitte,2015. Reference Source

[92] UK Government: The Digital Transformation Portfolio.London: UK Government,2019. Reference Source

[93] Sharma SC Sharma S Thakker A : Revolution in UK general practice due to COVID-19 pandemic: a cross-sectional survey. *Cureus.* 2020;12(8):e9573. 10.7759/cureus.9573 32913690 PMC7474564

[94] Palmer VJ Weavell W Callander R : The Participatory Zeitgeist: an explanatory theoretical model of change in an era of coproduction and codesign in healthcare improvement. *Med Humanit.* 2019;45(3):247–57. 10.1136/medhum-2017-011398 29954854 PMC6818522

[95] Purohit A Smith J Hibble A : Does telemedicine reduce the carbon footprint of healthcare? A systematic review. *Future Healthc J.* 2021;8(1):e85–e91. 10.7861/fhj.2020-0080 33791483 PMC8004323

[96] Tsagkaris C Hoian AV Ahmad S : Using telemedicine for a lower carbon footprint in healthcare: A twofold tale of healing. *J Clim Chang Health.* 2021;1:100006. 10.1016/j.joclim.2021.100006

[97] LeRouge C Ma J Sneha S : User profiles and personas in the design and development of consumer health technologies. *Int J Med Inform.* 2013;82(11):e251–e68. 10.1016/j.ijmedinf.2011.03.006 21481635

[98] Knight J Ross E Gibbons C : Unlocking Service Flow—Fast and Frugal Digital Healthcare Design. Design of Assistive Technology for Ageing Populations: Springer,2020;167:171–87. 10.1007/978-3-030-26292-1_9

[99] Glaser BG : The Constant Comparative Method of Qualitative Analysis*. *Soc Probl.* 2014;12(4):436–45.

[100] Glaser BG : The constant comparative method of qualitative analysis. *Soc Probl.* 1965;12(4):436–45. 10.2307/798843

[101] Salisbury C Lay-Flurrie S Bankhead CR : Measuring the complexity of general practice consultations: a Delphi and cross-sectional study in English primary care. *Br J Gen Pract.* 2021;71(707):e423–e31. 10.3399/BJGP.2020.0486 33824162 PMC8049201

[102] Greenhalgh T Shaw S Alvarez Nishio A : Remote care in UK general practice: baseline data on 11 case studies [version 1; peer review: awaiting peer review]. *NIHR Open Res.* 2022. 10.3310/nihropenres.13290.1 PMC7614213 36814638

