# Supplementary material for: Protocol: Remote care as the ‘new normal’?  Multi-site case study in UK general practice
Source: NIHR Open Res. 2022 Aug 8;2:46. [Version 1] doi: 10.3310/nihropenres.13289.1 (PMC10593351; doi:10.3310/nihropenres.13289.1)
Supplement: Peer review history

# Reviewer report: Reviewer response for version 1

**DOI:** 10.3310/nihropenres.14413.r29093

**Published:** 2023-05-02

**Authors:** Elaine C. Khoong

**Affiliations:** 1 University of California San Francisco, San Francisco, California, USA

Thank you for the opportunity to review the protocol for this large study that includes three main workstreams: (1) an in-depth study of 11 general practices across the UK and their approach to using remote health tools; (2) working with patients to co-design an ideal approach to provision of remote health care; and (3) focused interviews and dissemination to leaders and policy-makers to develop strategies that promote the best practices from workstreams 1 and 2.

 My comments are minor with some suggestions for ways to increase the impact of the study:

 # Terminology: I think if this protocol is written for just a UK audience, then this is probably fine, but if not, there are some terminologies that may be less familiar to other audiences (e.g., GP surgeries, online consultations – which is defined in the background but was initially confusing in the plain language summary to me).

 PLAIN LANGUAGE SUMMARY

 # Workstream 2: *“Many people experience barriers to accessing care when it is done through technology. This could be because they lack understanding of how to do it, don’t have the right equipment, can’t afford data, or…”* – I bristled a bit at this because it was very deficiency focused. It seemed to blame patients for their lack of understanding rather than stating that perhaps clinics were not inclusive in their design. The authors themselves later on note that a “deficiency framing overlooks the pervasive impact of multiple interacting social determinants.” Suggest reframing this in the plain language summary.

 # Workstream 2: In this summary and in the work itself, it could be beneficial to focus activities on how patients can achieve digital inclusion. (I like the definition from NDIA, based in the US: https://www.digitalinclusion.org/definitions/) Happy for authors to use another definition, but I think it helps provide a framing mechanisms for what domains need to be addressed to ensure digital inclusion.

 # Workstream 3: The authors do not include this in their work right now and it may not be a good fit given funding source, but as a US-based reviewer, I’d be interested in knowing enough about the context of different findings to understand how the extensive work the authors will do can be applied to different US contexts. If authors have the bandwidth and expertise to be explicit about how to apply these findings to other high-income countries with similar challenges, I suspect there would be great interest.

 WORKSTREAM 1 PROTOCOL

 # Table 1: In addition to qualitative description of practice demographics, it may be valuable to have a quantitative measure of an area-level SDOH indicator for patients (such as area deprivation index, which is used in the US) “assigned” to the practice (if that occurs in UK). It might be helpful to describe more generically how disadvantaged vs advantaged the patient population is.

 # Quality and safety of care approach: consider adding chart reviews or conducting root cause analyses of poor outcomes as a way to add some additional insight to any concerning outcomes. I noted the authors describe some intersecting work at three practices that focuses on consulting activity over a two-year period. I think identifying 5-10 poor outcomes in each practice and conducting a root cause analyses or chart review could provide some additional useful insights.

 # Workload, staff wellbeing approach: it wasn’t clear, but just wanted to make sure authors consider use of some of the existing validated burnout surveys to measure this (in addition the proposed interviews).

 # Sub-sample studies: the authors note a focus on three practices to evaluate the the front-desk and back-office work. Some rationale about why these three practices were chosen would be helpful. Same thing for the practices to be chosen for the climate impact evaluation.

 # General analytic approach: these detailed case studies would seem to be well-suited for coincidence analysis. The authors could take these detailed qualitative descriptions about practices at several points in team and identify its association with for example % patients that receive telephone triage, rates of patient satisfaction, or any other outcome. It may be helpful for informing understanding of what factors seemed to matter most in specific contexts.

Is the study design appropriate for the research question?

Yes

Is the rationale for, and objectives of, the study clearly described?

Yes

Are sufficient details of the methods provided to allow replication by others?

Yes

Are the datasets clearly presented in a useable and accessible format?

Not applicable

Reviewer Expertise:

Digital health, implementation science, chronic disease management and disparities, primary care, safety net systems

I confirm that I have read this submission and believe that I have an appropriate level of expertise to confirm that it is of an acceptable scientific standard.

# Response

**Published:** 2023-07-12

**Authors:** Trisha Greenhalgh

**Affiliations:** University of Oxford, UK

Thanks so much for this helpful report. We are almost at the stage of writing up our final report on this project. Rather than amending the protocol paper therefore, we'll take careful note of these comments when writing up our findings. In particular, we agree we framed the digital disparities theme negatively as a 'deficiency' in service users. Our data collected during RBD2 illustrates how services sometimes do manage to be more inclusive, and we'll make sure we provide a more balanced account!

# Reviewer report: Reviewer response for version 1

**DOI:** 10.3310/nihropenres.14413.r29094

**Published:** 2023-04-11

**Authors:** Kristin N. Ray

**Affiliations:** 1 University of Pittsburgh, Pittsburgh, Pennsylvania, USA

Thank you for the chance to review this manuscript detailing the protocol for a multi-site, mixed-methods case study of remote care in general practice. The protocol manuscript is clearly written, and motivates and details the proposed work well.

 The study rationale and objectives are clearly described. Specifically, the manuscript successfully articulates the value of understanding in rich detail how individual practices are successfully (and unsuccessfully) using one or more modalities of remote care by investigating a diverse set of general practices across the UK.

 The use of mixed-methods, multi-site case studies is appropriate and adequately detailed. The study will triangulate data from researchers-in-residence embedded in each practice, along with perspectives from patients, staff, and national-level stakeholders. The protocol also embraces an action research lens, thereby seeking to support practices as they continue to evaluate and innovate in their use of remote care.

 The protocol includes details to intentionally include individuals often marginalized by health care systems and often excluded from research. Specific details note strategies to recruit and interview individuals who may speak limited English, have lower digital literacy, experience substance use, or be unhoused. One detail that was not clear was the capacity of the research team to conduct interviews in languages other than English – are there specific other languages that individuals with limited English proficiency will be able to be interviewed in (and perhaps other languages where participation will not be able to be accommodated?).

 Strong patient and lay representation on the external advisory board as well as through workshops throughout the study period.

 Conclusion statement appropriately addresses the anticipated value and limitations of the proposed study.

Is the study design appropriate for the research question?

Yes

Is the rationale for, and objectives of, the study clearly described?

Yes

Are sufficient details of the methods provided to allow replication by others?

Yes

Are the datasets clearly presented in a useable and accessible format?

Not applicable

Reviewer Expertise:

Primary care researcher focused on impact of technology and telehealth on health care access, outcomes, and equity through quantitative and qualitative methods.

I confirm that I have read this submission and believe that I have an appropriate level of expertise to confirm that it is of an acceptable scientific standard.

# Reviewer report: Reviewer response for version 1

**DOI:** 10.3310/nihropenres.14413.r28751

**Published:** 2022-09-05

**Authors:** Andrew Turner

**Affiliations:** 1 Centre for Academic Primary Care, Population Health Sciences, University of Bristol, Bristol, UK; 2 National Institute for Health Research Applied Research Collaboration West (NIHR ARC West), University Hospitals Bristol and Weston NHS Foundation Trust, Bristol, UK

Thank you for the invitation to review this excellent protocol.

 The protocol describes a mixed-methods study exploring what the role of remote care (telephone, online, video) ought to be in UK general practice.

 The rationale for the research is clear and the design and methods are entirely appropriate. The protocol makes a strong case for the value of detailed practice level case-studies (workstream 1) alongside patient (workstream 2), and national stakeholder (workstream 3) perspectives to address the research questions.

 My only comments are all very minor:

- The plain English summary includes different words and phrases for the same thing: "general practices", "GP surgeries", "surgeries" and "practices". In the rest of the article, the sites are mostly called 'practices'. I suggest sticking to general practices (and, or, practices) all the way through.
- The description of Workstream 3 in the plain English summary reads as if it doesn't include any data collection, but in the rest of the protocol you explain that you intend to interview national stakeholders. I think this should be clarified in the summary.
- The diversity of recruited practices is good and characterising their digital maturity gives important context. A clarification question about digital maturity:- Given the pressures on general practice at the moment, I imagine there might be practices that are currently unable to do the things in levels 2 - 5 (described in Table 2), even though they want to and ordinarily would. I wasn't sure whether the capacity/headroom practices have to think about or change their services right now gets considered as part of their digital maturity (is it part of capability, readiness, or infrastructure?), or whether that's a different issue. Does being at a higher level entail that practices have the capacity to innovate at the moment?

Is the study design appropriate for the research question?

Yes

Is the rationale for, and objectives of, the study clearly described?

Yes

Are sufficient details of the methods provided to allow replication by others?

Yes

Are the datasets clearly presented in a useable and accessible format?

Not applicable

Reviewer Expertise:

Primary care, Digital health, Science and technology studies

I confirm that I have read this submission and believe that I have an appropriate level of expertise to confirm that it is of an acceptable scientific standard.
